# The Effectiveness of Combination Therapy for Treating Methicillin-Susceptible *Staphylococcus aureus* Bacteremia: A Systematic Literature Review and a Meta-Analysis

**DOI:** 10.3390/microorganisms10050848

**Published:** 2022-04-20

**Authors:** Sara Grillo, Mireia Puig-Asensio, Marin L. Schweizer, Guillermo Cuervo, Isabel Oriol, Miquel Pujol, Jordi Carratalà

**Affiliations:** 1 Department of Infectious Diseases, Bellvitge University Hospital, Bellvitge Institute for Biomedical Research (IDIBELL), Feixa Llarga s/n, 08907 L’Hospitalet de Llobregat, Spain; sara.grillo@bellvitgehospital.cat (S.G.); guillermo.cuervo@bellvitgehospital.cat (G.C.); mpujol@bellvitgehospital.cat (M.P.); jcarratala@bellvitgehospital.cat (J.C.); 2 Centro de Investigación en Red de Enfermedades Infecciosas (CIBERINFEC; CB21/13/00009), Instituto de Salud Carlos III, 28029 Madrid, Spain; 3 Department of Internal Medicine, University of Iowa Carver College of Medicine, Iowa City, IA 52242, USA; marin-schweizer@uiowa.edu; 4 Center for Access and Delivery Research and Evaluation, Iowa City VA Health Care System, Iowa City, IA 52246, USA; 5 Hospital Sant Joan Despí Moisés Broggi, Oriol Martorell 12, 08970 Sant Joan Despí, Spain; isabel.oriolbermudez@sanitatintegral.org; 6 Department of Medicine, University of Barcelona, 08007 Barcelona, Spain

**Keywords:** *Staphylococcus aureus*, methicillin-susceptible, bacteremia, combination therapy, meta-analysis

## Abstract

Background: This meta-analysis aims to evaluate the effectiveness of combination therapy for treating MSSA bacteremia. Methods: We searched Ovid MEDLINE, EMBASE, Cochrane CENTRAL, and clinicaltrials.gov for studies including adults with MSSA bacteremia. The monotherapy group used a first-line antibiotic active against MSSA and the combination group used a first-line antibiotic plus additional antibiotic/s. The primary outcome was all-cause mortality. Secondary outcomes included persistent bacteremia, duration of bacteremia, relapse, and adverse events. Random-effects models with inverse variance weighting were used to estimate pooled risk ratios (pRR). Heterogeneity was assessed using the *I*^2^ value and the Cochrane’s Q statistic. Results: A total of 12 studies (6 randomized controlled trials [RCTs]) were included. Combination therapy did not significantly reduce 30-day mortality (pRR 0.92, 95% CI, 0.70–1.20), 90-day mortality (pRR 0.89, 95% CI, 0.74–1.06), or any-time mortality (pRR 0.91, 95% CI, 0.76–1.08). Among patients with deep-seated infections, adjunctive rifampicin may reduce 90-day mortality (3 studies with moderate-high risk of bias; pRR 0.62, 95% CI, 0.42–0.92). For secondary outcomes, combination therapy decreased the risk of relapse (pRR 0.38, 95% CI, 0.22–0.66), but this benefit was not maintained when pooling RCTs (pRR 0.54, 95% CI, 0.12–2.51). Combination therapy was associated with an increased risk of adverse events (pRR 1.74, 95% CI, 1.31–2.31). Conclusions: Combination therapy not only did not decrease mortality in patients with MSSA bacteremia, but also increased the risk of adverse events. Combination therapy may reduce the risk of relapse, but additional high-quality studies are needed.

## 1. Introduction

*Staphylococcus aureus* is a leading cause of bloodstream infections, with an incidence of disease burden that has risen in recent decades due to the increase in healthcare procedures and the use of indwelling devices [1]. Additionally, *S. aureus* bacteremia (SAB) is associated with significant morbidity, mortality, and healthcare costs [2].

Despite the availability of active antibiotics against methicillin-susceptible *S. aureus* (MSSA) [3,4] and the sound clinical evidence that adherence to quality-of-care bundle interventions is associated with better outcomes [5], 30-day mortality is still high, ranging from 15 to 30% [6]. This poor prognosis of SAB has prompted clinicians to look for other strategies to improve patient outcomes, including the use of combination antibiotic therapies.

Previous in vitro and in vivo experimental studies have shown that certain combinations of antibiotics are associated with increased bactericidal activity, better intracellular and biofilm penetration, and a lower risk of developing antibiotic resistance during SAB treatment [7,8]. Thus, patients at the highest risk of mortality or SAB complications (those with prosthetic devices, endocarditis, or sepsis) might benefit the most from combination therapy.

To date, the effectiveness of combination therapies to improve SAB prognosis remains controversial. For methicillin-resistant *S. aureus* (MRSA), a recent meta-analysis has concluded that beta-lactam therapy combined with vancomycin or daptomycin does not reduce SAB mortality [9]. For MSSA, the evidence is even more scarce and derived from studies that have important limitations. Previous meta-analyses have grouped different end-points of mortality together (all-cause, 30-day, in-hospital mortality) [10,11,12], included both *S. aureus* and streptococcal endocarditis [10], or focused only on rifampin combinations while mixing methicillin-resistant (MRSA) and MSSA infections [11]. In all, it remains unclear which subgroup of patients benefits the most from combination therapy, or which antibiotic combinations are the most effective for reducing SAB mortality.

To address these gaps in knowledge and to gain insights into the effectiveness of combination therapies for treating MSSA bacteremia—which has been associated with less mortality than MRSA [13]—we conducted this meta-analysis. We assessed whether different populations (e.g., patients with endocarditis) might benefit from this strategy in terms of mortality and persistent bacteremia. We also determined the risk of adverse events that may limit the generalized use of combined therapies.

## 2. Methods

### 2.1. Search Strategy

This meta-analysis followed the Preferred Reporting Items for Systematic and Meta-Analysis (PRISMA) Statement, and it was registered on PROSPERO (number CRD42020163104). We searched for studies evaluating the efficacy of combination antibiotic therapies for treating MSSA bacteremia compared to monotherapy.

A health sciences librarian conducted the search by using subject headings and keywords for Ovid MEDLINE, EMBASE, and Cochrane CENTRAL (Wiley) from database inception until 29 January 2021 (Supplementary material). We searched clinicaltrials.gov to detect unpublished studies. The reference lists of included articles were reviewed to identify additional studies.

### 2.2. Eligibility Criteria

We applied the following inclusion criteria: (1) the study population consisted of adults with MSSA bacteremia. Studies with methicillin-susceptible and resistant *S. aureus* strains were included if 10–15% of the study population had MRSA infections. This threshold was chosen to assure that ≥90% of study population received empiric therapy against MSSA; (2) the study was a randomized controlled trial (RCT), a quasi-experimental (non-randomized) trial, or an observational study. Non-RCTs were included to improve generalizability; (3) the monotherapy group used an active antibiotic against *S. aureus*; the combination group used the antibiotic administered in monotherapy plus additional antibiotic/s; and (4) the study reported mortality rates, persistent bacteremia, or duration of bacteremia. We excluded non-research articles, conference abstracts, and in vitro and animal studies.

### 2.3. Outcomes

The primary outcome was all-cause mortality, which included 30-day, 90-day, or any-time mortality. Secondary outcomes were persistent bacteremia or duration of bacteremia, relapse, adverse events, and development of drug resistance.

### 2.4. Data Extraction and Quality Assessment

Titles and abstracts were screened for eligibility. Two independent researchers reviewed full-text articles and abstracted data using a standardized abstraction form. Data collected included study design and population, number of patients, *S. aureus* resistance to methicillin, antibiotics evaluated, and outcomes. We also recorded variables associated with better SAB survival: echocardiography use, infectious diseases (ID) consultation, and adherence to quality-of-care bundle interventions [5,14]. Disagreements between reviewers were resolved by consensus. When two articles had overlapping study populations, the more comprehensive was included.

The risk of bias for the association between combination therapy and mortality was assessed using the Cochrane tool for RCTs [15] and the Robins-I tool for observational studies [16].

### 2.5. Statistical Analysis

We used random-effects models with inverse variance weighting to estimate pooled risk ratios (pRR). Studies with no events (outcomes) in either study arm were omitted from the meta-analysis. For RCTs, intention-to-treat data were used whenever possible. We grouped studies that assessed 28-day mortality with those reporting 30-day mortality, and reported combined pRRs. If different endpoints for mortality were reported, we preferably evaluated 30- and 90-day mortalities. We assessed heterogeneity using the Cochrane Q statistic and the *I*^2^ test. Publication bias was assessed through visual inspection of funnel plots and the Egger test. For mortality, we performed subgroup analyses based on a priori selected variables: study design, antibiotic in combination, source of infection, and indication of the combination therapy (persistent bacteremia vs. early therapy). Deep-seated infections included osteoarticular infections, endocarditis, deep-seated abscesses, or foreign body infections. Statistical analyses were performed using the Cochrane Review Manager (RevMan, version 5.3), Comprehensive Meta-Analysis Version 3 software (Englewood, NJ, USA), and Excel 2007.

## 3. Results

**Figure 1:**
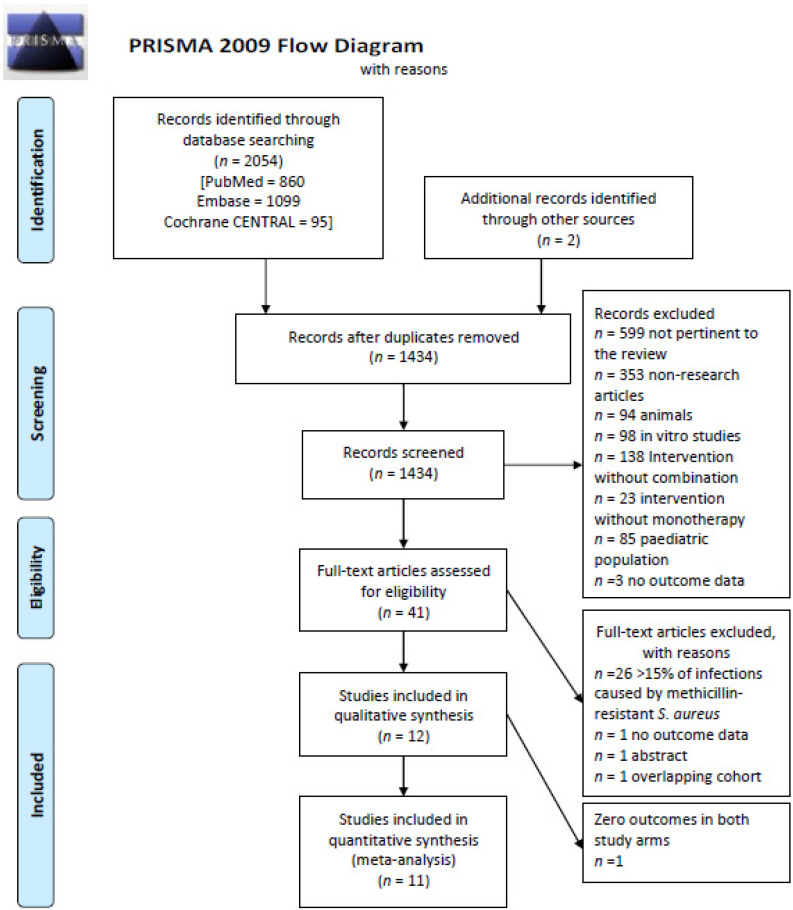
Flowchart of the selection of the studies.

We screened 2054 articles and included 12 studies that met the inclusion criteria (Figure 1). One study had zero outcomes in both study arms, and it was included in the systematic literature review but not in the meta-analysis [17]. Table 1 summarizes the characteristics of included studies. There were six RCTs [17,18,19,20,21,22] and six observational studies [23,24,25,26,27,28]. Six studies were multicentric (≥2 hospitals) [18,19,20,22,23,24] and five studies were published before 2000 [17,20,21,25,28].

### 3.1. Study Groups

In the monotherapy group, beta-lactams (antistaphylococcal penicillins, first-generation cephalosporins) were the main antibiotics used. Two studies including ≤11% of MRSA bacteremias also used vancomycin, teicoplanin, linezolid, or daptomycin [18,23]. In four studies, the monotherapy group comprised different types of antibiotics against MSSA and included a few patients receiving an antibiotic that was not the optimal standard of care (i.e., vancomycin, ceftriaxone, or clindamycin) [19,23,24,28].

Five studies evaluated the combination therapy with aminoglycosides [17,20,21,25,28], three with rifampicin [18,19,24], two with daptomycin [22,26], and one with vancomycin [27]. One study described two separate analyses for patients who received combination therapy with levofloxacin and a post-hoc analysis of patients with a deep-seated focus who received additional rifampicin [19]. One study was classified in the rifampicin group despite including a small proportion of patients (19%) who received fosfomycin in combination [23]. Also, two studies including >70% of patients with a deep-seated focus used rifampicin, fluoroquinolones, or aminoglycosides along with the antibiotics in the monotherapy/combination groups [19,24].

### 3.2. Start of Combination Therapy

Among studies with available data, eight reported early administration of combination therapy. That is, combination therapy was initiated ≤72 h after blood sample collection, ≤96 h after starting active treatment [18,22,25,26,27], or after the suspicion of endocarditis [17,20,21]. In two studies evaluating the combination with rifampicin, this treatment was started later, within seven [19] or fourteen days [23] after blood sample collection.

### 3.3. Sources of Infection

Most studies evaluated patients with *S. aureus* bacteremia, regardless of the source of infection. However, studies assessing the combination with aminoglycosides focused on patients with endocarditis [17,20,21,25,28]. Two studies evaluating the combination with rifampicin and one post-hoc analysis restricted their analyses to patients with deep-seated infections or with foreign devices [19,23,24]. The remaining five studies included a variable proportion of patients with endocarditis (4% to 23%) [18,19,22,23,26] or with prosthetic materials (6% to 34%) [18,19,22,26]. In one study evaluating the combination therapy with daptomycin, 7% of patients had pneumonia [22] (Table 1; Supplementary Table S1).

### 3.4. Compliance with Good Clinical Practices

One RCT evaluating the combination therapy with daptomycin reported that all patients received the optimal standard of care, with removal of intravascular devices, performance of echocardiogram, and ID consultation [22]. The remaining studies did not list complete compliance with these quality-of-care indicators (Supplementary Table S1). Eight reported that all or nearly all patients (>79%) [17,18,19,23,24,25,26,28] were followed by an ID specialist, but only four mentioned rates of source control as part of SAB management [18,19,23,26] or indications for an echocardiogram [21,26].

### 3.5. Risk of Bias

All observational studies had moderate-high risk of bias when assessed by the Robins-I tool (Supplementary Figures S1 and S2). Confounding bias was frequent, probably because combination therapy was commonly administered in more severely ill patients. Four studies used appropriate methods to control for confounding factors of mortality (Table 1) [23,24,26,27].

Among the RCTs, 36% of the participants in a double-blind study assessing rifampicin knew they were receiving the study drug [18].

Six studies assessed duration of bacteremia or persistent bacteremia [18,20,21,22,26,27], but only four obtained follow-up blood cultures at scheduled times [18,20,21,22]. The two best designed RCTs performed their analyses with competing risk analysis [18] or with a time-to-event analysis [11].

### 3.6. Effectiveness of Combination Therapy

Eleven studies were included in the meta-analysis: all reported mortality rates, three duration of bacteremia [20,22,27], and two persistent bacteremia [21,26]. Five studies reported relapse rates [17,21,22,23,24] and five drug-related adverse events [18,20,21,22,27]. One study assessed the development of drug resistance during treatment [18]. Study outcomes are displayed in Supplementary Table S2.

#### 3.6.1. Mortality

**Figure 2:**
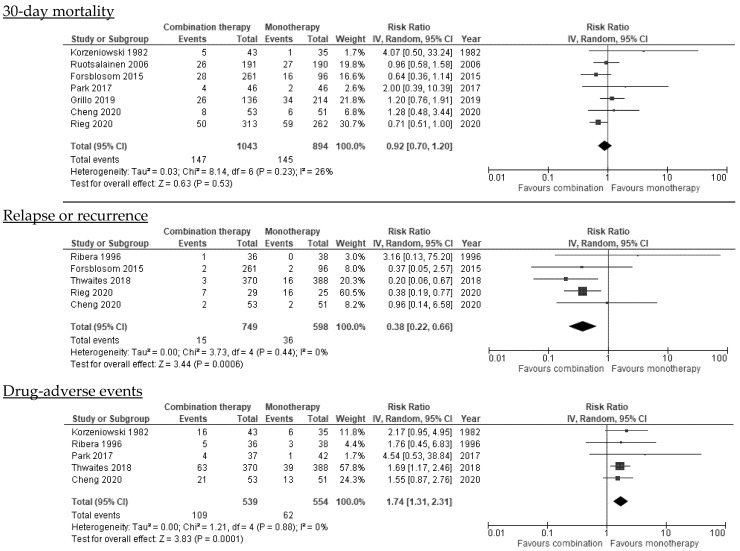
Effectiveness of combination therapy on main outcomes.

All-cause mortality was 14.4% (216/1503) in the combination group and 16.1% (215/1339) in the monotherapy group. Pooled results did not show a significant reduction in the risk of 30-day (seven studies; pRR 0.92, 95% CI, 0.70–1.20, *I*^2^ = 26%), 90-day (six studies; pRR 0.89, 95% CI, 0.74–1.06, *I*^2^ = 27%), or any-time mortality (11 studies; pRR 0.91, 95% CI, 0.76–1.08, *I*^2^ = 0%) in patients who received combination therapy compared to monotherapy (Figure 2; Table 2). These results were consistent when studies with late or unknown start of combination therapy or when studies published before 2000 were excluded. Heterogeneity was low. The funnel plot for 30-day and any-time mortality was asymmetrical and there was evidence of publication bias (Supplementary Figures S3–S5).

Subgroup analyses performed separately for RCTs and observational studies did not yield different results, but heterogeneity was moderate-high among observational studies (30-day mortality: *I*^2^ = 42%; 90-day mortality: *I*^2^ = 68%) (Table 2). To investigate causes of heterogeneity, we pooled observational studies that were adjusted for confounding factors and RCTs with well-balanced characteristics between study groups. The heterogeneity decreased and results were consistent with the main findings, with no reductions in 30- and 90-day mortality with combination therapy (pRR 1.04, 95% CI, 0.67–1.60; *I*^2^ = 0% and pRR 0.96, 95% CI, 0.78–1.19; *I*^2^ = 13%, respectively).

We next hypothesized that the source of SAB might influence the effectiveness of combination therapy. The meta-analysis of three studies including patients with deep-seated infections suggested that combination therapy with rifampicin might be associated with decreased 90-day mortality (pRR 0.62, 95% CI, 0.42–0.92), but the heterogeneity was high: *I*^2^ = 73% (Table 2). These studies had a moderate-high risk of bias, and they were of lower quality. Also, the pooled analysis of studies including patients with endocarditis did not demonstrate that the combination therapy with aminoglycosides reduced any-time mortality (four studies: pRR 1.17, 95% CI, 0.64–2.16; *I*^2^ = 0%). These studies focused on patients with native valve endocarditis, in particular right-sided endocarditis (30–100% of participants) in drug users. Other subgroup analyses according to the source of infection were not possible.

#### 3.6.2. Duration of Bacteremia

Three studies [20,22,27] reported the mean or median duration of bacteremia, but only two provided the standard deviations needed to calculate the standardized mean difference [20,27]. Therefore, we could not meta-analyze these data.

The role of combination therapy to increase the rapid killing of MSSA is unclear. With regard to aminoglycosides, one RCT of 78 patients with native valve endocarditis (68.8% right-sided) showed that adding gentamycin during the first two weeks of therapy was associated with faster eradication of SAB (mean 2.9 vs. 3.8 days) [20]. However, other RCTs assessing the combination with rifampicin [18] or daptomycin (mean 2.0 vs. 1.7 days) [22], and a small cohort study evaluating vancomycin (median 2.8 vs. 2.2 days) [27], did not find an association between combination therapy and duration of bacteremia.

#### 3.6.3. Persistent Bacteremia

Two studies assessed persistent bacteremia, namely positive blood cultures for ≥3 days or at 4 days [21,26], and they showed contradictory results. One RCT of 90 patients with MSSA right-sided native valve endocarditis did not find that combination therapy with aminoglycosides during the first seven days of therapy reduced persistent bacteremia (2.6% [1/38] vs. 0% [0/36]) [21]. This study had limited statistical power for this outcome. Conversely, an observational study found that persistent bacteremia was more frequent using daptomycin in combination compared to monotherapy (26.7% [35/131] vs. 9.1% [18/197]; *p* < 0.001) [26]. However, this result may reflect unadjusted imbalances between study groups, with more severely ill patients receiving the combination therapy.

#### 3.6.4. Relapse or Recurrence

Five studies, three of which explored rifampicin combinations, reported SAB relapse by demonstrating the bacteriological recovery of *S. aureus* with the same resistance pattern from the bloodstream [22,23,24] or from a sterile site during follow-up [18]. One study required the comparison of *S. aureus* isolates by phage-typing [21]. The time point of relapse measurement varied across studies: within 90 days [22,24] or 6 months [21,23]. One study used the term “recurrence,” defined as the isolation of the same strain of *S. aureus* from a sterile site after seven days of improvement [18].

The overall relapse/recurrence rates were 2.0% (15/749) for patients with combination therapy and 6.0% (36/598) for those who received monotherapy. Pooled results suggested that combination therapy was significantly associated with a decreased risk of relapse (five studies; pRR 0.38, 95% CI, 0.22–0.66; *I*^2^ = 0%), but there was possible publication bias (Supplementary Figure S6). Similar conclusions were obtained for studies evaluating rifampicin (three studies; pRR 0.32, 95%CI CI, 0.18–0.58; *I*^2^ = 0%), although two studies had a moderate-high risk of bias [23,24]. Importantly, subgroup analyses performed on better designed RCTs showed differing results, with no significant association between combination therapy and relapse (three studies; pRR 0.54, 95% CI, 0.12–2.51), although heterogeneity was moderate (*I*^2^ = 46%) (Table 2).

### 3.7. Drug-Related Adverse Events

Combination therapy was significantly associated with adverse events (five studies; pRR 1.74, 95% CI, 1.31–2.31; *I*^2^ = 0%) (Table 2), in particular with nephrotoxicity, which was mainly assessed in studies including nephrotoxic drugs (four studies; pRR 1.81, 95% CI, 1.17–2.79; *I*^2^ = 0%). Conclusions did not change after excluding studies published before 2000 (three studies; pRR 1.69, 95% CI, 1.24–2.30; *I*^2^ = 0%) [18,22,27]. The funnel plot showed possible evidence of publication bias (Supplementary Figure S7). One study evaluated *Clostridioides difficile* diarrhea and concluded that there were no significant differences between the study groups [24]. Other adverse events were rarely reported (Table 1).

### 3.8. Effectiveness per Type of Antibiotic in Combination

Detailed descriptions of studies and pooled analyses per type of antibiotic (aminoglycosides and rifampicin) are summarized in the Supplementary material.

## 4. Discussion

In this meta-analysis, we found that patients with MSSA bacteremia who received combination therapy not only did not present a lower risk of all-cause mortality but also presented an increased risk of adverse events. Although our data suggest that combination therapy reduces the risk of relapse and that patients with deep-seated infections or implanted foreign devices may benefit from combinations with rifampicin, additional studies are needed to better define recommendations for its use.

Focusing on specific combinations, the evidence does not favor combination therapy with aminoglycosides. The studies included were mostly performed in patients with right-sided endocarditis, but none of them demonstrated that the addition of aminoglycosides decreased mortality rates. It is possible that patients who receive aminoglycosides experience faster clearance of MSSA bacteremia [20], but this benefit does not affect mortality, and it is associated with a greater risk of nephrotoxicity. Thus, our results support current guidelines [29,30] that do not recommend the use of low-dose gentamycin for treating SAB, even in patients with native valve endocarditis.

Rifampicin is an appealing option as a combination therapy. It has good activity against *S. aureus* and its biofilm, being useful for treating hardware-associated infections. Our results suggest that combinations including rifampicin may reduce 90-day mortality and relapse in bacteremic patients with deep-seated infections and implanted foreign body devices. This evidence is mainly derived from observational studies with a moderate-high risk of bias, but the results are plausible given rifampicin’s mechanism of action. Indeed, combination therapy with rifampicin is recommended in *S. aureus* prosthetic joint infections and prosthetic valve endocarditis [30,31]. However, there is no evidence supporting its use in bacteremic patients who do not have these sources of infection. First, a well-conducted RCT (the ARREST trial) including 758 patients with a low proportion of prosthetic device infections did not show that adding rifampicin provided any benefit in reducing mortality or shortening the duration of bacteremia [18]. Second, the early administration of rifampicin during the bacteremic phase of the infection may promote the development of resistance [32]. The appropriate time to start rifampicin combinations in bacteremic patients is an unresolved issue.

Few of the studies assessed other combination therapies. According to one small cohort study [27], beta-lactams plus vancomycin did not reduce SAB mortality or the duration of bacteremia. However, the use of beta-lactams with vancomycin may play a role as empirical therapy for covering both methicillin-susceptible and resistant strains. The use of vancomycin for MSSA bacteremia is associated with poorer outcomes than beta-lactams [33]. Consequently, some clinicians might opt to use this empirical combination when there is a suspicion of staphylococcal bacteremia and *S. aureus* susceptibility is not known [4].

Similarly, the early combination of beta-lactams with daptomycin is not warranted. Neither of the included studies found significant differences in mortality, duration of bacteremia, or relapse when adding daptomycin within the first four days of SAB treatment [22,26]. These results contrast with reports describing successful outcomes when using ceftaroline plus daptomycin as salvage therapy, especially in patients with persistent MRSA bacteremia [34,35,36]. Although an experimental model of infective endocarditis suggested that cloxacillin plus daptomycin have a synergistic effect [37], further clinical studies are needed.

Finally, reports or case-series have described the use of two beta-lactams such as ertapenem plus cefazolin as salvage therapy for persistent MSSA bacteremia [38,39]. However, there are no RCTs assessing the effectiveness of this combination for SAB treatment.

This meta-analysis shows that the use of combination therapies comes at a cost of an increased risk of adverse events. In the ARREST trial [18], for example, the addition of rifampicin was associated with antibiotic adverse events and drug–drug interactions, even though 11% of screened patients were not enrolled because of pre-existing liver disease and the risk of drug interactions. Interestingly, none of the studies evaluated the risk of colonization for multi-drug resistant bacteria and only one study mentioned no differences in *C. difficile* diarrhea between study arms. Future studies should evaluate both effectiveness and adverse events; conditions that may impact the extended use of antibiotic combinations.

Our meta-analysis has certain limitations. The studies we included differed in sources of *S. aureus* bacteremia, outcome definitions, type and duration of combinations, and the dose of antibiotics evaluated. When possible, we pooled studies with similar characteristics to provide more reliable results, but study populations were heterogeneous. Furthermore, the impact of race and gender was not examined in subgroup analyses, and these factors may have affected treatment response. Some observational studies had selection biases that were often not properly addressed and this might have biased the results towards the null. However, we performed subgroup analyses of studies that adjusted for confounding factors and well-balanced RCTs yielding similar conclusions to our overall pooled analysis. Finally, most studies did not report data regarding adherence to good-quality-of-care indicators [5] or the rates of source control. To improve the quality of research, future studies should use standardized endpoints (probably earlier timepoints of mortality to optimize antibiotic combination assessment), focus on high-risk patients (e.g., patients with implanted foreign devices), and describe the compliance with good clinical practices to ensure the best management of SAB [40].

In conclusion, the currently available data do not support the use of combination therapy to reduce mortality in all patients with MSSA bacteremia. Further, this therapeutic strategy is associated with an increased risk of drug-related adverse events. Patients with deep-seated infections or implanted foreign devices may benefit from a late start of combinations with rifampicin, but more studies are needed to define recommendations on its use. Well-designed multicenter trials with large samples and with patient-risk stratification for evaluating the combination therapy are urgently needed.

## Figures and Tables

**Table 1:** Main characteristics of the studies included.

Author, Year Country ^a^	Study Design; n° of Hospitals	Study Population	Monotherapy Group	Antibiotic/s Added in the Combination Group ^b^	Reason for Starting the Combination Therapy	Duration of the Combination Therapy ^c^	Sample Size (Combination/Monotherapy Groups)	Outcomes Assessed					Adjusted for the Following Confounders:
								Mortality	Duration of Bacteremia	Persistent Bacteremia	Relapse	Adverse Events	
Watanakunakorn, 1977, US	Cohort study; 1	SAB and endocarditis (possibly MSSA)	OXA/PEN/CFZ	GEN 3-5 mg/kg/day	Patients who had been treated recently	2–3 weeks	40 (15 vs. 25)	Yes, death during the 6 weeks of treatment	No	No	No	Nephrotoxicity	None
Abrams, 1979, US	RCT; 1	Intravenous drug users with MSSA bacteremia and endocarditis	OXA or CFZ	GEN 80 mg/8 h	After randomization	2 weeks PP	25 (12 vs. 13)	Yes, all-cause mortality (end-point of assessment not reported)	No	No	No	Nephrotoxicity, drug-induced hepatitis, leukopenia, drug fever and rash.	None, study groups well-balanced
Rajashekaraiah, 1980, US	Cohort study; 1	MSSA bacteremia and endocarditis	OXA/PEN/CFZ 4–6 weeks	GEN 4.5 mg/kg/day	GEN added because of lack of prompt clinical response Combination started ≤ 48 h after starting treatment	At least 7 days	33 (21 vs. 12)	Yes, all-cause mortality (end-point of assessment not reported)	No	No	No	No	None
Korzeniowski, 1982, US	RCT; 1	SAB and native valve endocarditis (possibly MSSA)	PEN 6 weeks	GEN 3 mg/kg/8 h	After randomization	2 weeks PP	78 (35 vs. 43)	Yes, 30-day	Yes	No	No	Nephrotoxicity	None
Ribera, 1996, Spain	RCT; 1	Intravenous drug users with right-sided MSSA endocarditis	OXA 14 days	GEN 1 mg/kg/8 h	After randomization	7 days PP	74 (36 vs. 38)	Yes, death during treatment	No	Yes	Yes	Nephrotoxicity	None, study groups well-balanced
Ruotsalainen, 2006, Finland	RCT; 13	MSSA bacteremia	BL+/− AMG (If endocarditis)+/−RIF	LVX 500 mg once or b.i.d according to weight.	After randomization	42 days (28–58)	381 (191 vs. 190)	Yes, 30 and 90-days	No	No	No	Yes, liver enzyme elevations, *Clostridoides difficile*, and allergic reactions evaluated.	None, study groups well-balanced
Ruotsalainen, 2006, Finland	Post-hoc analysis of a prospective cohort; 13	MSSA bacteremia with deep seated infection	BL+/− AMG (If endocarditis) +/−LVX	LVX+ RIF 450 or 600 mg/day	NR	Unknown	331 (265 vs. 66)	Yes, 90-day	No	No	No	No	NR
Forsblom, 2015, Finland	Retrospective cohort; 13	MSSA bacteremia and deep infection focus	**OXA (58%)**, CXM, CRO, VAN or CLI ^d^ +/−FLQ/AMG	RIF 450 or 600 mg/day	NR	Short (1–13 days) Long (≥14 days)	357 (261 vs. 96)	Yes, 30 and 90-days	No	No	Yes	No	Age, severity of illness, ID consultation, endocarditis, ultimately-rapidly fatal disease
Park, 2017, Korea	Retrospective matched cohort; 1	MSSA bacteremia	BL	VAN ^e^	NR, but combination was an empirical treatment	2.8 days (2.1–3.8)	92 (46 vs. 46)	Yes, 30-day	Yes	No	No	Nephrotoxicity	Charlson score, Pitt score, white blood cell count
Thwaites, 2018, UK	RCT; 29	Patients with SAB who have received ≤ 96 h of active antibiotic treatment (94% MSSA; 6% MRSA bacteremia)	**OXA (83%)**, VAN or TEC	RIF 600 or 900 mg/day	After randomization	12.6 days (6.0–13.2)	708 (370 vs. 388)	Yes, 90-day	Yes	No	Yes, recurrence	Yes, all type. Serious adverse events, drug-modifying adverse events, drug interactions	None, study groups well-balanced
Grillo, 2019, Spain	Post-hoc analysis of a prospective cohort; 1	Patients with MSSA bacteremia who survived > 48h	BL	DAP 10 mg/kg/day	NR; but combination was administered for ≥72 h and started within the first 4 days of treatment	9 days (4–15)	350 (136 vs. 214)	Yes, 7, 30 and 90-days	No	Yes	No	No	Age, Pitt score, source of infection
Rieg, 2020, Germany	Post-hoc analysis of a prospective cohort; 2	SAB with deep-seated infection (89% MSSA; 11% MRSA bacteremia)	**OXA**, DAP, VAN, LIN	**RIF** (450 mg/12 h) [77.3%] or FOS (5 g/8 h iv)	NR; combination started within 14 days after SAB onset	23 days (13–33)	578 (313 vs. 265)	Yes, 30 and 90-days	No	No	Yes	No	Age, Charlson, severe sepsis
Cheng, 2020, Canada	Double-blind RCT; 2	Patients with MSSA bacteremia with ≤72 h from first positive blood culture	BL	DAP 6 mg/kg/day	After randomization	5 days PP	104 (53 vs. 51)	Yes, 30 and 90-days	Yes	No	Yes	Nephrotoxicity, hepatotoxicity, rhabdomlyolisis within 5 days of enrollment	None, study groups well-balanced

^a^ Abbreviations: SAB, *Staphylococcus aureus* bacteremia; RCT, randomized controlled trial; PP, per protocol; IVDU, intravenous drug users; OXA, oxacillin; PEN, penicillin; CFZ, cefazolin; GEN, gentamycin; BL, beta-lactams; AMG, aminoglycosides; RIF, rifampicin; LVX, levofloxacin; CMX, cefuroxime; CRO, ceftriaxone; CLI, clindamycin; FLQ, fluoroquinolones; DAP, daptomycin; VAN, vancomycin; TEC, teicoplanin; LIN, linezolid; FOS, fosfomycin; MSSA, methicillin-susceptible *S. aureus*. NR: not reported. ^b^ Antibiotic added to the monotherapy backbone. ^c^ Data presented as median (IQR) otherwise specified. ^d^ When multiple types of antibiotics are listed, the most frequently administered is highlighted in bold and the percentage is indicated in parenthesis. ^e^ Dose of vancomycin was not reported.

**Table 2:** Summary of the subgroup analyses performed.

	Subgroup Analyses	N°. of Studies	References	N°. of Patients (Combination/Monotherapy)	Pooled Risk Ratio (95% CI)	*I*^2^ Test
30-day mortality	All studies	7	[19,20,22,23,24,26,27]	1043/894	0.92 (0.70–1.20)	26%
	RCTs	3	[19,20,22]	287/276	1.08 (0.70–1.67)	0%
	Observational studies	4	[23,24,26,27]	756/618	0.85 (0.60–1.22)	42%
	Adjusted observational studies + well-balanced RCTs	3	[19,22,27]	290/287	1.04 (0.67–1.60)	0%
	Early administration of combination therapy	4	[20,22,26,27]	278/346	1.31 (0.88–1.95)	0%
	After excluding studies published before 2000	6	[19,22,23,24,26,27]	1000/859	0.88 (0.69–1.13)	18%
90-day mortality	All studies	6	[18,19,22,23,24,26]	1336/1179	0.89 (0.74–1.06)	27%
	RCTs	3	[18,19,22]	632/611	0.93 (0.72–1.20)	0%
	Observational studies	3	[23,24,26]	704/568	0.86 (0.60–1.22)	68%
	Adjusted observational studies + well-balanced RCTs	5	[18,19,22,23,26]	1075/1083	0.96 (0.78–1.19)	13%
	After excluding studies with late start of combination therapy	3	[18,22,26]	577/635	1.07 (0.84–1.37)	0%
	All participants with deep-seated infections (rifampicin) ^a^	3	[19,23,24]	833/420	0.62 (0.42–0.92)	73%
Any-time mortality ^b^	All studies	11	[18,19,20,21,22,23,24,25,26,27,28]	1503/1339	0.91 (0.76–1.08)	0%
	RCTs	6	[18,19,20,21,22,25]	732/696	1.01 (0.78–1.31)	0%
	Observational studies	5	[23,24,26,27,28]	771/643	0.86 (0.64–1.15)	25%
	After excluding studies with late start of combination therapy	7	[18,20,21,22,25,26,27]	723/766	1.09 (0.85–1.41)	0%
	After excluding studies published before 2000	7	[18,19,22,23,24,26,27]	1388/1229	0.89 (0.74–1.08)	5%
	All participants with endocarditis (aminoglycosides) ^c^	4	[20,21,25,28]	115/110	1.17 (0.64–2.16)	0%
	≥30% left-sided endocarditis (aminoglycosides)	3	[20,25,28]	79/72	1.12 (0.60–2.11)	0%
Relapse ^d^	All studies	5	[18,21,22,23,24]	749/598	0.38 (0.22–0.66)	0%
	Excluding Thwaites study that used a different definition (recurrence)	4	[21,22,23,24]	379/210	0.45 (0.24–0.83)	0%
	RCTs	3	[18,21,22]	459/477	0.54 (0.12–2.51)	46%
	Observational studies	2	[23,24]	290/121	NA	
Drug adverse-events ^d^	Any type of antibiotic adverse-event	5	[18,20,21,22,27]	539/554	1.74 (1.31–2.31)	0%
	After excluding studies published before 2000	3	[18,22,27]	460/482	1.69 (1.24–2.30)	0%
	Nephrotoxicity or AKI	4	[20,21,22,27]	169/166	1.81 (1.17–2.79)	0%

Abbreviations: AKI, acute kidney injury; CI, confidence interval; RCT, randomized controlled trial; NA, not applicable. ^a^ All these studies compared the use of beta-lactams in monotherapy with beta-lactams plus rifampicin. ^b^ When different definitions of mortality were available for one study (e.g., 30-day mortality, 90-day mortality, mortality during antibiotic treatment), 30-day mortality was preferably chosen for pooled analyses. We hypothesized that at later time points, the evaluation of treatment-related effectiveness might be less accurate given the increasing effect of patient’s baseline comorbidities on mortality. ^c^ All these studies compared the use of beta-lactams in monotherapy with beta-lactams plus gentamicin. They primarily focused on patients with native valve endocarditis, in particular right-sided endocarditis (30-100% of participants) in drug users. ^d^ None of these studies adjusted their analyses when evaluating the outcome relapse or adverse event.

## Data Availability

Not applicable.

## Supplementary Materials

The following supporting information can be downloaded at: https://www.mdpi.com/article/10.3390/microorganisms10050848/s1, Figure S1: Risk of bias for observational studies; Figure S2: Risk of bias for randomised controlled trials; Figure S3: Funnel plot for 30-day mortality; Figure S4: Funnel plot for 90-day mortality; Figure S5: Funnel plot for any-time mortality; Figure S6: Funnel plot for relapse or recurrence; Figure S7: Funnel plot for drug adverse-events; Table S1: Detailed description of study population; Table S2: Outcomes. References [41,42] cited in Supplementary Materials.
