# Supplementary material for: The Effectiveness of Combination Therapy for Treating Methicillin-Susceptible Staphylococcus aureus Bacteremia: A Systematic Literature Review and a Meta-Analysis
Source: Microorganisms. 2022 Apr 20;10(5):848. doi: 10.3390/microorganisms10050848 (PMC9145429; doi:10.3390/microorganisms10050848)
Supplement: Supplementary file 1 [file microorganisms-10-00848-s001.zip › microorganisms-1671464-supplementary.pdf]

Effectiveness of combination therapy for treating methicillin-susceptible *Staphylococcus aureus* bacteraemia: a systematic literature review and meta-analysis

Sara Grillo*, Mireia Puig-Asensio*, Marin L. Schweizer, Guillermo Cuervo, Isabel Oriol, Miquel Pujol and Jordi Carratalà

Supplementary material

Index

Methods

Search strategy Page 2

Results

Sub-analysis by antibiotic groups Page 5

Supplementary, Table S1 Page 9

Supplementary, Table S2 Page 11

Supplementary Figures

Figure S1. Risk of bias for observational studies. Page 13

Figure S2. Risk of bias for randomised controlled trials. Page 14

Figure S3. Funnel plot for 30-day mortality. Page 15

Figure S4. Funnel plot for 90-day mortality. Page 16

Figure S5. Funnel plot for any-time mortality. Page 17

Figure S6. Funnel plot for relapse or recurrence. Page 18

Figure S7. Funnel plot for drug adverse-events. Page 19

References Page 20

Search strategies

Ovid MEDLINE

1. Staphylococcus aureus/
2. exp Methicillin-Resistant Staphylococcus aureus/
3. staphylococcus aureus.tw,kw.
4. s aureus.tw,kw.
5. mrsa.tw,kw.
6. mssa.tw,kw.
7. mrsab.tw,kw.
8. mssab.tw,kw.
9. 1 or 2 or 3 or 4 or 5 or 6 or 7 or 8
10. exp Bacteraemia/
11. exp Endocarditis, Bacterial/
12. bacter?emia*.tw,kw.
13. bloodstream infection*.tw,kw.
14. blood stream infection*.tw,kw.
15. bsi.tw,kw.
16. bsis.tw,kw.
17. endocarditis.tw,kw.
18. 10 or 11 or 12 or 13 or 14 or 15 or 16 or 17
19. Drug Therapy, Combination/
20. ((combination* or combine* or combining or combo$1 or second line) adj2 (therapy or therapies or therapeutic* or treatment* or regimen* or antibiotic* or anti-biotic* or antimicrobial* or anti-microbial* or group* or arm$1)).tw,kw.
21. ((synergist* or synergy) adj3 (therapy or therapies or therapeutic* or treatment* or regimen* or antibiotic* or anti-biotic* or antimicrobial* or anti-microbial* or combination* or combo$1 or activity)).tw,kw.
22. (standard* of care adj3 (combination* or combine* or combining or combo$1 or second line)).tw,kw.
23. (adjunctive or concomitant or escalated therap*).tw,kw.
24. 19 or 20 or 21 or 22 or 23
25. 9 and 18 and 24

EMBASE

#27. #10 AND #20 AND #26
#26. #21 OR #22 OR #23 OR #24 OR #25
#25. adjunctive:ti,ab,kw OR concomitant:ti,ab,kw OR 'escalated therap*':ti,ab,kw
#24. ((synergist* OR synergy) NEAR/3 (therapy OR therapies OR therapeutic* OR treatment* OR regimen* OR antibiotic* OR 'anti biotic*' OR antimicrobial* OR 'anti microbial*' OR combination* OR combo$ OR activity)):ti,ab,kw
#23. ((synergist* OR synergy) NEAR/3 (therapy OR therapies OR therapeutic* OR treatment* OR regimen* OR antibiotic* OR 'anti biotic*' OR antimicrobial* OR 'anti microbial*' OR combination* OR combo$ OR activity)):ti,ab,kw
#22. ((combination* OR combine* OR combining OR combo$ OR 'second line') NEAR/2 (therapy OR therapies OR therapeutic* OR treatment* OR regimen* OR antibiotic* OR 'anti biotic*' OR antimicrobial* OR 'anti microbial*' OR group* OR arm$)):ti,ab,kw

#21. 'combination drug therapy'/de
#20. #11 OR #12 OR #13 OR #14 OR #15 OR #16 OR #17 OR #18 OR #19
#19. endocarditis:ti,ab,kw
#18. bsis:ti,ab,kw
#17. bsi:ti,ab,kw
#16. 'blood stream infection*':ti,ab,kw
#15. 'bloodstream infection*':ti,ab,kw
#14. bacter$emia*:ti,ab,kw
#13. 'bacterial endocarditis'/exp
#12. 'bloodstream infection'/exp
#11. 'bacteraemia'/exp
#10. #1 OR #2 OR #3 OR #4 OR #5 OR #6 OR #7 OR #8 OR #9
#9.mssab:ti,ab,kw
#8.mrsab:ti,ab,kw
#7.mssa:ti,ab,kw
#6.mrsa:ti,ab,kw
#5.'s aureus':ti,ab,kw
#4.'staphylococcus aureus':ti,ab,kw
#3.'methicillin susceptible staphylococcus aureus'/exp
#2.'methicillin resistant staphylococcus aureus'/exp
#1.'staphylococcus aureus'/de

Cochrane CENTRAL (Wiley)

#1 MeSH descriptor: [Staphylococcus aureus] explode all trees
#2 ("staphylococcus aureus"):ti,ab,kw
#3 ("s aureus"):ti,ab,kw
#4 (mrsa):ti,ab,kw
#5 (mssa):ti,ab,kw
#6 (mrsab):ti,ab,kw
#7 (mssab):ti,ab,kw
#8 {OR #1-#7}
#9 MeSH descriptor: [Bacteraemia] explode all trees
#10 MeSH descriptor: [Endocarditis, Bacterial] explode all trees
#11 (bacter*mia):ti,ab,kw
#12 ("bloodstream infection"):ti,ab,kw
#13 ("bloodstream infections"):ti,ab,kw
#14 ("blood stream infection"):ti,ab,kw
#15 ("blood stream infections"):ti,ab,kw
#16 (bsi):ti,ab,kw
#17 (bsis):ti,ab,kw
#18 (endocarditis):ti,ab,kw
#19 {OR #9-#18}
#20 MeSH descriptor: [Drug Therapy, Combination] this term only
#21 (((combination* or combine* or combining or combo or combos or "second line") NEAR/2 (therapy or therapies or therapeutic* or treatment* or regimen* or antibiotic* or "anti biotic" or "anti biotics" or antimicrobial* or "anti microbial" or "anti microbials" or group* or arm OR arms))):ti,ab,kw
#22 ((synergist* or synergy) NEAR/3 (therapy or therapies or therapeutic* or treatment* or regimen* or antibiotic* or "anti biotic" or "anti biotics" or antimicrobial* or "anti microbial" or "anti microbials" or combination* or combo or combos or activity)):ti,ab,kw

#23 (("standard of care" or "standards of care") NEAR/3 (combination* or combine* or combining or combo or combos or "second line")):ti,ab,kw
#24 (adjunctive or concomitant or "escalated therapy" or "escalated therapies" or "escalated therapeutic" or "escalated therapeutics"):ti,ab,kw
#25 {OR #20-#24}
#26 #8 AND #19 AND #25

All database results were exported to EndNote and duplicates were removed using a multi-step process.

Results

Detailed descriptions of studies and pooled analyses per type of antibiotic

Combination with aminoglycosides

Study characteristics

Five studies (3 RCTS and 2 cohort studies) [1][2][3][4][5] comprising a total of 210 patients (128 combination group; 122 monotherapy) compared the use of beta-lactams monotherapy with beta-lactams plus aminoglycosides. These studies had small sample sizes and were published more than 2 decades ago, most of them before 1985 [1][2][3][4].

All but except one study [1] focused on patients with native valve endocarditis, in particular right-sided endocarditis in drug users. They evaluated the combination with gentamycin. However, the dose and the duration of gentamycin therapy varied across studies, usually ranging from 1 to 3 mg/kg of body weight every 8 hours for a total duration of 7 up to 14 days.

Mortality

None of these studies showed a reduction in mortality with combination therapy. However, mortality rates were evaluated at different time points: Two studies did not define the time point of assessment [2][3], two reported mortality during antibiotic treatment [1][5], and one reported 30-day mortality (among others) [4]. Death at any time point occurred in 14.1% (18 of 128) in the combination group and 12.3% (15 of 122) in the monotherapy group. One study had zero deaths in both arms [2]. When the remaining 4 studies were pooled together, combined therapy with aminoglycosides was not associated with a significant reduction of mortality (pooled RR 1.17, 95% CI 0.64–2.16; Table 2 within the manuscript). There was no significant heterogeneity between included studies (I^2=0%, p=0.60).

Duration or persistent bacteraemia

One RCT of 78 patients with native valve endocarditis (68.8% right-sided) showed that patients who received additional gentamycin during the first 2 weeks of therapy had faster eradication of *S.aureus* bacteraemia [4]. Another RCT of 90 patients with MSSA right-sided native valve endocarditis [5] did not find that combination therapy with for the first 7 days of therapy was associated with any significant benefit in terms of persistent bacteraemia and relapse [5]. However, it should be noted that this later study had limited statistical power for these outcomes.

Adverse events

Only two studies reported detailed information about nephrotoxicity, and thus, we could not meta-analyze these data [4][5]. However, nephrotoxicity was probably more likely to occur in patients receiving adjunctive therapy with aminoglycosides (17.1% monotherapy group vs 37.2% combination group and 7.9% vs 13.9%, respectively) [4][5].

Combination with rifampicin

Study characteristics

Four studies comprising 2,011 patients (1,203 combination group; 808 monotherapy) compared the use of beta-lactams with the combination therapy with rifampicin [6][7][8][9]. Two non-randomised studies and one post-hoc analysis of a RCT focused on patients with deep-seated infections [6][8][9] whereas the single RCT involved all focuses of *S. aureus* bacteraemia [7], and only 40% of study participants had a deep-seated focus and <8% of prosthetic devices or hardware.

The dose and duration of the combined treatment with rifampicin was varied, usually ranging from 600 to 900 mg/day and administered for more than 12 days. However, one study accepted a lower dose of rifampicin (450 mg once daily for patients under 50 kg) and included a small proportion of patients who received a short-course of rifampicin (between 1 and 13 days) [6].

Mortality

Only 2 studies described 30-day mortality and could not be meta-analyzed [6][8].We next examined 90-day mortality, which might provide a less accurate estimate of the true effectiveness of combination therapy. The pooled analysis of 4 studies [6][8][9][10] showed that the combination therapy did not reduce 90-day mortality (pRR 0.74, 95% CI 0.48–1.13). The test for heterogeneity was statistically significant (p=0.003, I^2=78%). When the single study that did not focus on deep-seated infections [7] was excluded from the analysis, the combination therapy was associated with a reduced risk of 90-day mortality (pRR 0.62, 95% CI 0.42–0.92), but the heterogeneity was still high (p=0.03, I^2=78%); Table 2 within the manuscript.

Duration bacteraemia

The RCT from Thwaites et al (ARREST) did not find that rifampicin shortened the duration of bacteraemia within the first 7 days of follow-up [7].

Relapse/recurrence

Three studies evaluated this outcome. Relapse was evaluated within 90 days of follow-up in one study [6] and within 180 days in the other [8]. The third one evaluated recurrence of microbiologically proven *S. aureus* infection after 7 days of clinical improvement [7]. The incidence of overall relapse/recurrence rate was 1.8% (12 of 660) in the combination group and 6.7% (34 of 509) in the monotherapy group. When these 3 studies were pooled together, combination therapy was significantly associated with a decreased risk of relapse (pRR 0.32, 95%CI CI, 0.18–0.58; I^2=0%). Of note, however, 2 studies had moderate-high risk of bias.

Adverse events

According to Thwaites et al, patients in the rifampicin group had more frequently antibiotic or drug-modifying adverse events by week 12 (17% vs 10%; p=0.004), predominantly gastrointestinal disorders (2.7% vs 1.8%) and drug interactions (6% vs 2%). Two rifampicin patients (0.5%) experienced hepatic adverse events, both with prior liver disease. Additionally, 2 out of 270 patients (0.7%) who received rifampicin, developed resistance to this drug during the follow-up.

Combination with daptomycin

Study characteristics

Two studies (1 RCT and 1 cohort study) comprising 454 patients (189 combination group; 265 monotherapy) compared the use of beta-lactams with the combination therapy with

daptomycin[11][12]. These studies addressed patients with SAB and < 15% of patients with endocarditis, osteoarticular infections, and implanted foreign materials.
The standard therapy was either cloxacillin or cephazolin. In the cohort study, however, other beta-lactams active against MSSA (i.e., meropenem or piperazillin-tazobactam) were also considered appropriate. The dose of daptomycin was lower in the RCT than in the cohort study (6 mg/kg/day versus 10 mg/kg/day, respectively). Daptomycin was given for a total of 5 days (RCT) to a median of 9 days (cohort study).

Mortality

The overall 30-day rate among the two studies was 18.0% (34/189) in the combination group and 15.1 % (40/265) in the monotherapy group.

Duration bacteraemia

The RCT did not find any significant reduction in the median duration of bacteraemia with the use of the combination therapy compared to the monotherapy (3.06 days vs 3.0 days; p=0.77)[11]. In contrast, the observational study concluded that persistent bacteraemia was more frequent in the combination group compared to the monotherapy group (26.7% [35/131] vs 9.1% [18/197], respectively; p<0.001)[12]. However, this result might reflect unadjusted imbalances between study groups, with more severely ill patients receiving the combination with daptomycin.

Relapse

The RCT did not find that receiving combination therapy was associated with lower likelihood of relapsed bacteraemia within 30 or 90 days (1.9% vs 0%; 3.8% vs 3.9%, respectively).

Adverse events

According to the RCT, there were no significant differences in the proportion of patients who developed renal failure (47.7% vs 28.9%), hepatoxicity (3.8% vs 5.9%) or rhabdomyolysis (0% vs 5.9%) within 5 days of enrollment between the combination and the monotherapy groups.

Combination with Levofloxacin

One single multicentric RCT assessed the combination therapy with levofloxacin (191 patients combination group; 190 monotherapy)[9]. Although this study included adult patients with SAB within 7 days of sampling, > 80% of patients had deep infections (e.g., infected prosthetic material, endocarditis, osteomyelitis).

Levofloxacin was dosed at 500 mg/day and was given for a long period of time (median of 42 days. Deep-seated infections and endocarditis could be treated with an additional therapy with an aminoglycosides or rifampicin apart from the study drug; this was more frequent in the monotherapy group.

Mortality

Combination therapy with levofloxacin did not decrease 28-day (14% combination vs 14% monotherapy) or 90-day mortality (18% combination vs 21% monotherapy).

Adverse events

Although data was not shown, authors reported no significant differences regarding the number of patients with liver enzyme elevations, *Clostridioides difficile* diarrhea or allergic reactions between the study groups.

Combination with vancomycin

One retrospective study including 46 patients in each study arm evaluated the role of combination therapy with vancomycin as empirical treatment; before knowing the antibiotic susceptibility of *S. aureus* [13]. Not surprisingly, the median duration of the combination therapy was as short as 2.8 days. Of note, the dose of vancomycin and the number of patients with endocarditis, osteoarticular infections, and implanted foreign materials were not reported.

Outcomes

The empirical use of vancomycin in combination with a beta-lactam was not associated with decreased 30-day mortality (adjusted HR 1.54, 95% CI 0.25–9.53) or duration of bacteraemia (2.8 days vs 2.2 days). More patients in the combination group developed acute renal failure (10.8% vs 2.4%). However, all these results should be interpreted with caution given the limited statistical power of the analyses.

Supplementary Table S1. Detailed description of study population

Author, year country [a]	IV drug users, No (%)	Endocarditis, No (%) [% only right-sided]	Patients with prosthetic material [b], n (%)	Renal impairment, No (%) [% dialysis]	ID consult	Polimicrobial bacteraemia	Quality-of-care indicators[c]
Watanakunakorn, 1977, United States	Yes, 7 (17.5%)	Yes, all study population [30.0%]	NR, but 2 patients with prosthetic valve (5%)	Probably included, but NR	Yes, per study protocol	No	A, C
Abrams, 1979, United States	Yes, all study population	Yes, all study population [76.9%]	NR	NR	Yes, per study protocol	Unknown	C
Rajashekaraiah, 1980, United States	Yes; 14 (42.4%, all patients with right endocarditis)	Yes, all study population [42.4%]	NR	Yes, 1 patient	Yes, per study protocol	No	C
Korzeniowski, 1982, United States	Yes; 48 (61.5%)	Yes, all study population [68.8%]	No	NR	Unknown	No	A
Ribera, 1996, Spain	Yes, all study population	Yes, all study population [100%]	NR	Yes, NR	Unknown	No	A, D
Ruotsalainen, 2006, Finland	NR	Yes, 70 (18%) [NR]	129 (34%)	59 (16%)	Yes, per study protocol	No	A, B, C
Ruotsalainen, 2006, Finland (post-hoc analysis)	NR	Yes, 70 (21%) [NR]	NR	45 (14%)	Yes, per study protocol	No	A, B, C
Forsblom, 2015, Finland	NR	Yes, 62 (17.3%)	Yes (NR)	Yes (NR)	Yes, 91%	Unknown	C
Park, 2017, Korea	NR	NR	NR	21 (23%)	Unknown	No	No
Thwaites, 2018, United Kingdom	NR	Yes, 33 (4%). Prosthetic valve endocarditis 7 (1%).	Infected implanted vascular device 36 (5%). Prosthetic valve and joint 14 (2%)	198 (18%)	Yes, per study protocol	No	A, B, C

Grillo, 2019, Spain	NR	Yes, 31 (8.9%) [NR]	Cardiac devices 16 (5.8%); ostheoarticular prosthesis 25 (9%)	98 (28.2%)	Yes, per study protocol	Unknown	B, C, D
Rieg, 2020, Germany	NR	Yes, 133 (23%) [NR]	378 (65.4%) (orthopedic, vascular graft, pacemaker and cardiac prosthetic valve)	NR	Yes, 79%	Unknown	B, C
Cheng, 2020, Canada	NR	Yes, 12 (11.5%) [NR]	Intravascular 14, (13.5%); extravascular foreign body 9 (8.7%); prosthetic cardiac valve 9 (8.7%)	NR	Yes	No	A, B, C,D

[a]Abbreviations: ID, infectious diseases; IV, intravenous; SAB, *Staphylococcus aureus* bacteraemia; MSSA, methicillin-susceptible *S. aureus*; NR, not reported.

[b]Prosthetic material: cardiac devices (including pacemaker and cardiac prosthetic valve), orthopedic devices and vascular graft.

[c]Quality-of-care indicators; A: follow-up blood culture obtained at a scheduled time; B: source control; C: infectious diseases consult; D: indications for the performance of an echocardiography reported.

Supplementary Table S2. Outcomes

<table>
<tr><th colspan="16">Monotherapy vs combination therapy</th></tr>
<tr><th>Author, year</th><th colspan="2">30-day mortality, n (%)</th><th colspan="2">90-day mortality, n (%)</th><th colspan="2">Other mortality[a], n (%)</th><th colspan="2">Persistent bacteraemia[b], n (%)</th><th colspan="2">Duration of bacteraemia, mean days (SD)</th><th colspan="2">Relapse[c], n (%)</th><th colspan="2">Adverse events[d], n (%)</th><th>Conclusions</th></tr>
<tr><td colspan="16">Adjuntive Aminoglycoside</td></tr>
<tr><td>Watanakunakorn, 1977</td><td colspan="2">NR</td><td colspan="2">NR</td><td>10/25 (40)</td><td>6/15 (40)</td><td colspan="2">NR</td><td colspan="2">NR</td><td colspan="2">NR</td><td colspan="2">No differences (NR)</td><td>No significant differences</td></tr>
<tr><td>Abrams, 1979</td><td colspan="2">NR</td><td colspan="2">NR</td><td>0/12 (0)</td><td>0/13 (0)</td><td colspan="2">NR</td><td colspan="2">NR</td><td colspan="2">NR</td><td>0/12 (0)</td><td>0/13 (0)</td><td>No significant differences</td></tr>
<tr><td>Korzeniowski, 1982</td><td>1/35 (2.9)</td><td>5/43 (11.6)</td><td colspan="2">NR</td><td colspan="2">NR</td><td colspan="2">NR</td><td>3.75 (1.3)</td><td>2.85[e] (1.25)</td><td colspan="2">NR</td><td>6/35 (17.1)</td><td>16/43 (37.2)</td><td>Combination reduced the bacteraemia duration. No significant differences in mortality. More nephrotoxicity with combination therapy.</td></tr>
<tr><td>Rajashekaraiah,1980</td><td colspan="2">NR</td><td colspan="2">NR</td><td>3/12 (25.0)</td><td>5/21 (23.8)</td><td colspan="2">NR</td><td colspan="2">NR</td><td colspan="2">NR</td><td colspan="2">NR</td><td>No significant differences</td></tr>
<tr><td>Ribera, 1996</td><td colspan="2">NR</td><td colspan="2">NR</td><td>1/38 (2.6)</td><td>2/36 (5.6)</td><td>1/38 (2.6)</td><td>0/36 (0)</td><td colspan="2">NR</td><td>0/38 (0)</td><td>1/36 (2.7)</td><td>3/38 (7.9)</td><td>5/36 (13.9)</td><td>No significant differences</td></tr>
<tr><td colspan="16">Adjuntive Rifampicin</td></tr>
<tr><td rowspan="2">Ruotsalainen, 2006 (post-hoc analysis)</td><td colspan="2" rowspan="2">NR</td><td>25/66 (37.9)</td><td>44/265 (16.6)</td><td colspan="2" rowspan="2">NR</td><td colspan="2" rowspan="2">NR</td><td colspan="2" rowspan="2">NR</td><td colspan="2" rowspan="2">NR</td><td colspan="2" rowspan="2">NR</td><td rowspan="2">Combination decreased 90-day mortality</td></tr>
<tr><td colspan="2">3.06; 95%CI (1.69–5.54), p < 0.001</td></tr>
<tr><td rowspan="2">Forsblom, 2015</td><td>16/96 (16.7)</td><td>28/261 (10.7)</td><td>25/96 (26.0)</td><td>41/261 (15.7)</td><td colspan="2" rowspan="2">NR</td><td colspan="2" rowspan="2">NR</td><td colspan="2" rowspan="2">NR</td><td>2/96 (2.0)</td><td>2/261 (0.7)</td><td colspan="2" rowspan="2">NR</td><td rowspan="2">Combination decreased 90-day mortality</td></tr>
<tr><td colspan="2">OR 0.60 (0.31-1.17), p=0.130</td><td colspan="2">OR 0.53 (0.30-0.93), p = .026</td><td colspan="2">OR 0.40 (0.06-2.89), p=0.349</td></tr>
</table>

Rieg, 2020	59/262 (22.5)	50/313 (16.0)	96/258 (37.2)	94/307 (30.6)	NR		NR		NR		16/255 (6.3)	7/297 (2.4)	NR		Combination decreased 90-day mortality in patients with implanted foreign devices
	P=0.054		Adjusted HR 0.69 (0.47-1.02) p = 0.061								P=0.031				
Thwaites, 2018	NR		56/388 (14.4)	56/370 (15.1)	17/388 (4.3)	25/370 (6.7)	NR		No differences (NR)		NR		39/388 (24.2)	63/370 (27.2)	No significant differences
			HR 1.10 (0.76-1.60), p =0.60		HR 1.60 (0.86-2.95), p = .13								HR 1.78 (1.20-2.65), p=0.004		
**Adjuntive Levofloxacin**															
Ruotsalainen, 2006	27/190 (14.2)	26/191 (13.6)	39/190 (20.5)	34/191 (17.8)	NR		NR		NR		3 patients in total (differences between groups NR)		No differences (NR)		No significant differences
	OR 1.05 (0.59-1.88), p=0.87		OR 1.19 (0.72-1.99), p=0.50												
**Adjuntive Vancomicin**															
Park, 2017	2/46 (4.3)	4/46 (8.7)	NR		NR		NR		2.58 (0.82)	3.03[e] (0.97)	NR		1/42 (2.4)	4/37 (10.8)	No significant differences
	HR 1.54 (0.249-9.532), p= 0.642														
**Adjuntive Daptomycin**															
Grillo, 2019	34/214 (15.9)	26/136 (19.1)	47/214 (22.0)	37/136 (27.2)	15/214 (7.0)	12/136 (8.8)	18/197 (9.1)	35/131 (26.7)	NR		NR		NR		No significant differences
			Adjusted HR 0.9 (0.57-1.43), p=0.67				P < .001								
Cheng, 2020	6/51 (11.8)	8/53 (15.1)	9/51 (17.6)	10/53 (18.9)	NR		NR		1.65	2.04	2/51 (3.9)	2/53 (3.8)	13/51 (25.5)	21/53 (39.6)	No significant differences

Abbreviations: NR, not reported; SD, standard deviation.

[a] Time of assessment: 7-day in Ribera and Grillo, 14-day in Thwaites, 6-weeks in Watanakunakorn, NR in Abrams and Rajashekaraiah.

[b] Assessment at 4 days in Ribera, at 72h after starting antibiotic treatment in Grillo.

[c] Time of assessment: 90-day in Forsblom and Cheng, 6-months in Ribera and Rieg.

[d] Nephrotoxicity in Watanakunakorn, Abrams, Korzeniowski, Ribera, Park and Cheng; antibiotic-modifying adverse events in Thwaites; *C. difficile* infection, allergic reaction or liver enzyme elevation in Ruotsalainen.

[e] For studies that reported the duration of bacteraemia as a median [inter-quartile range (IRQ) or range], we estimated the mean as devised by Wan et al [14].

Supplementary Figures

Figure S1. Risk of bias for observational studies.

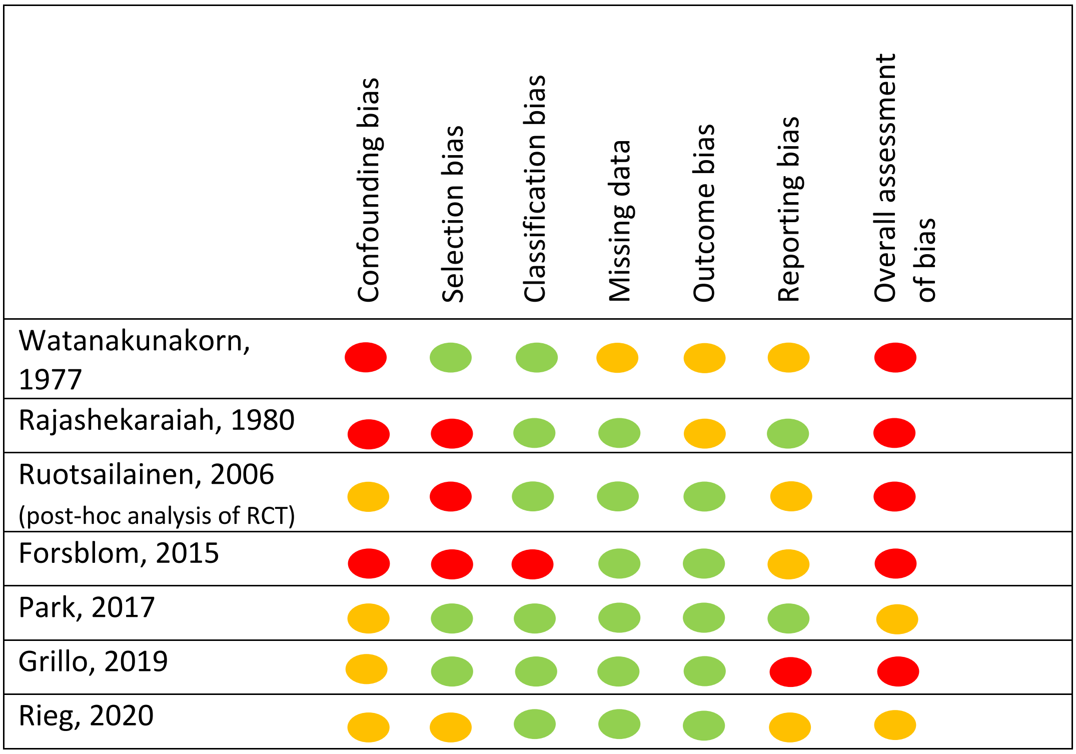

Figure S2. Risk of bias for randomised controlled trials.

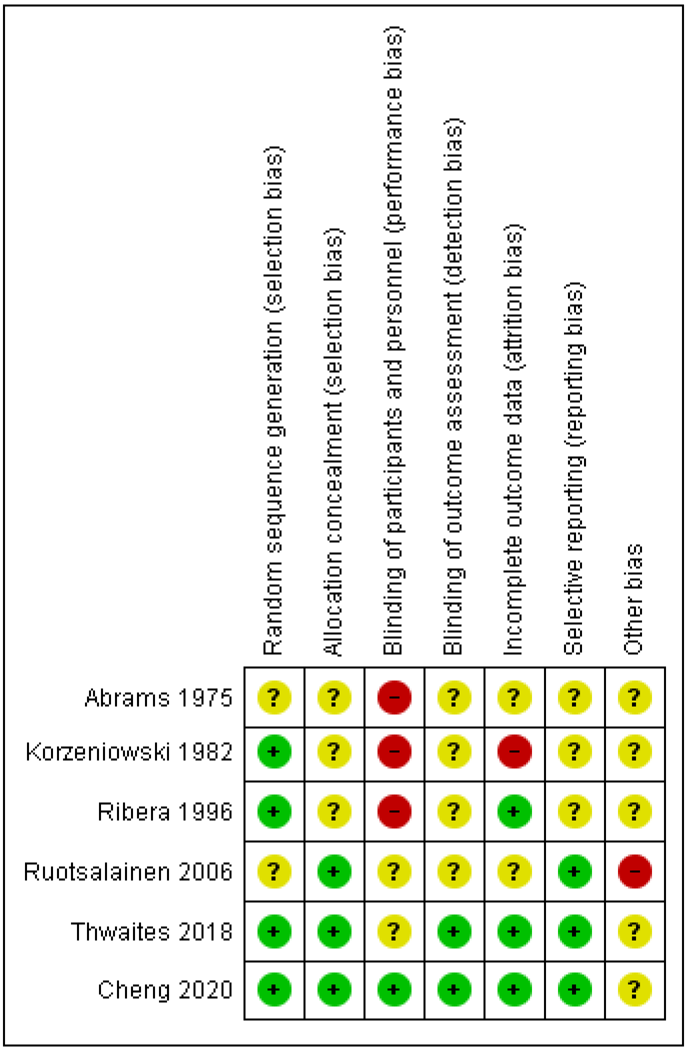

Figure S3. Funnel plot for 30-day mortality.

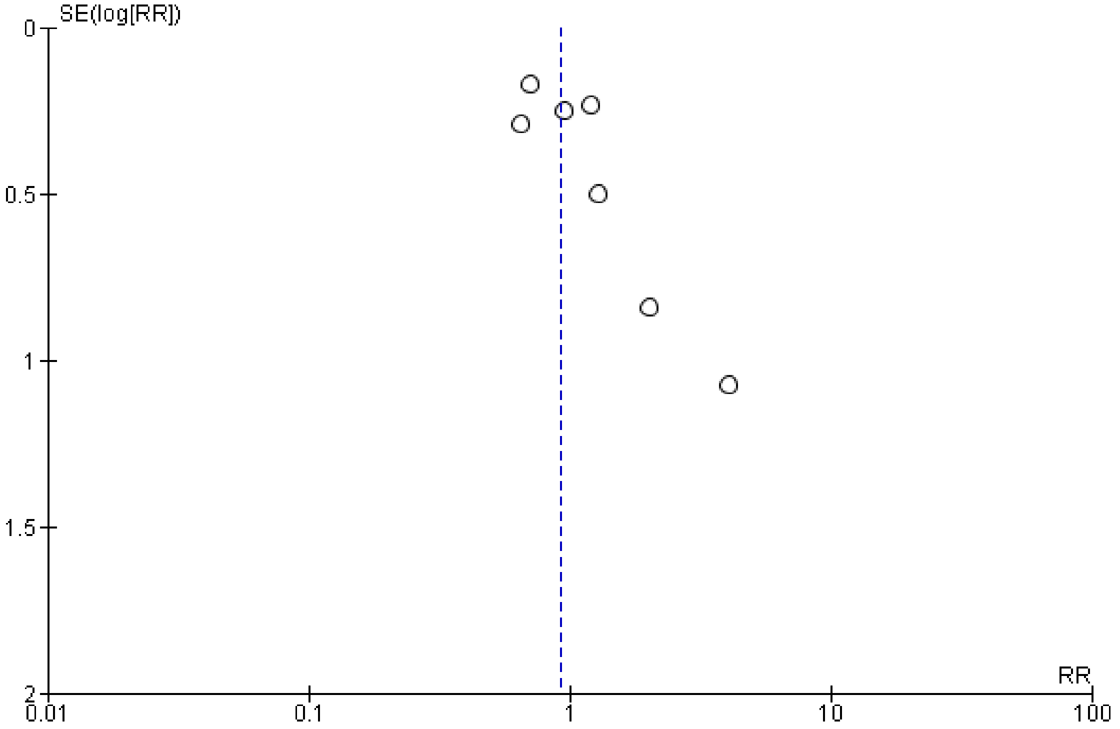

Figure S4. Funnel plot for 90-day mortality.

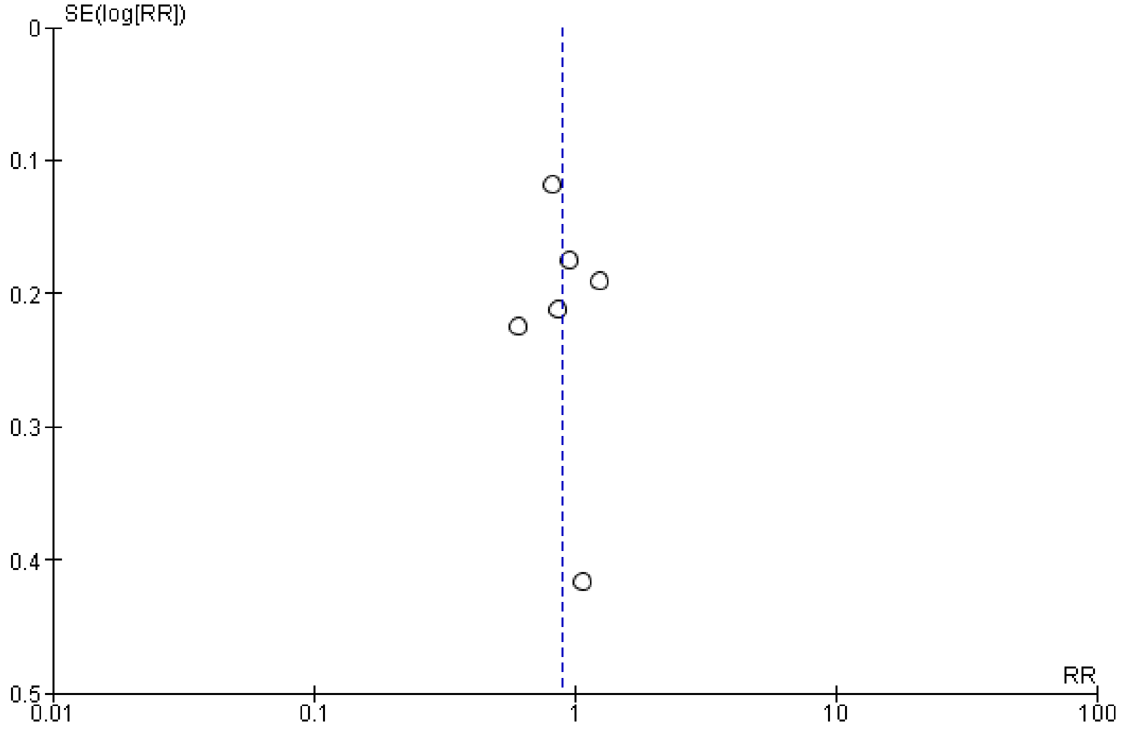

Figure S5. Funnel plot for any-time mortality.

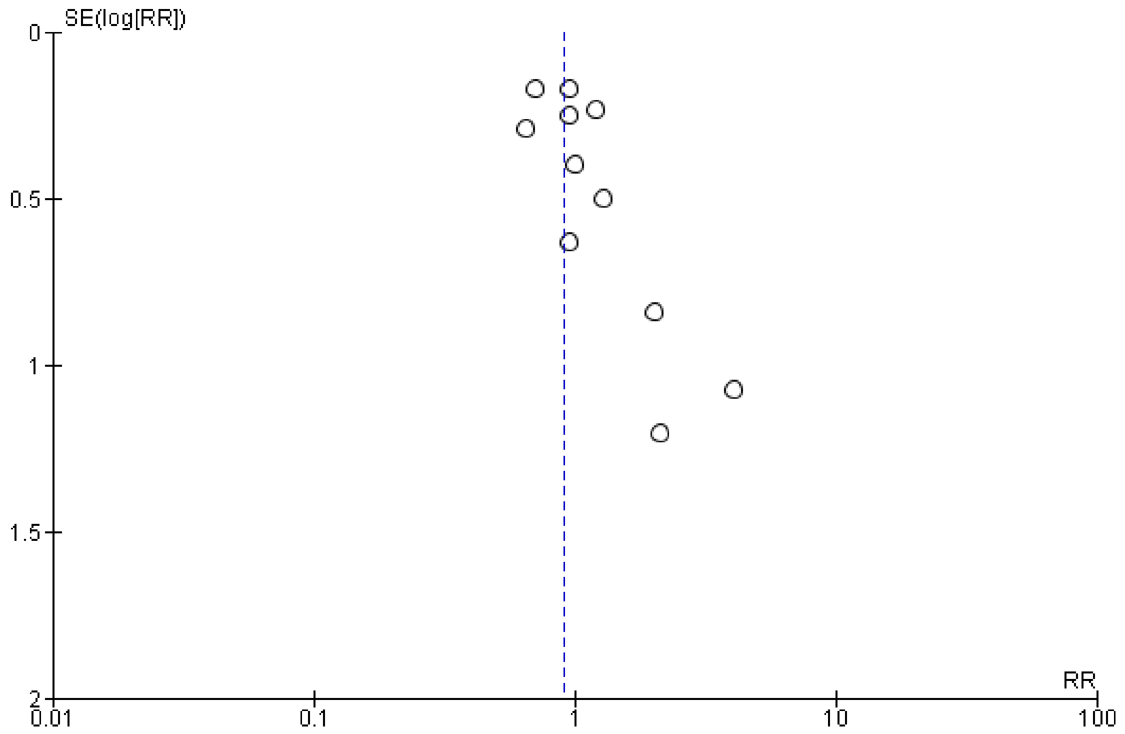

*The Egger test also showed some evidence for publication bias (p=0.048)

Figure S6. Funnel plot for relapse or recurrence

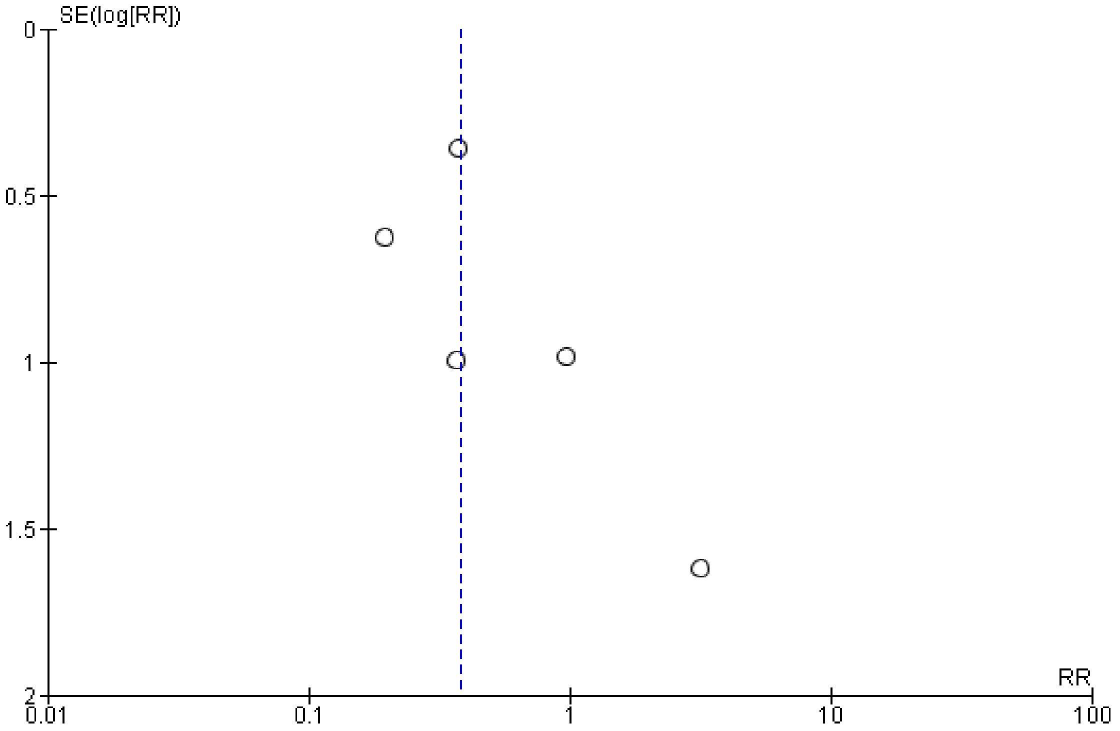

Figure S7. Funnel plot for drug adverse-events

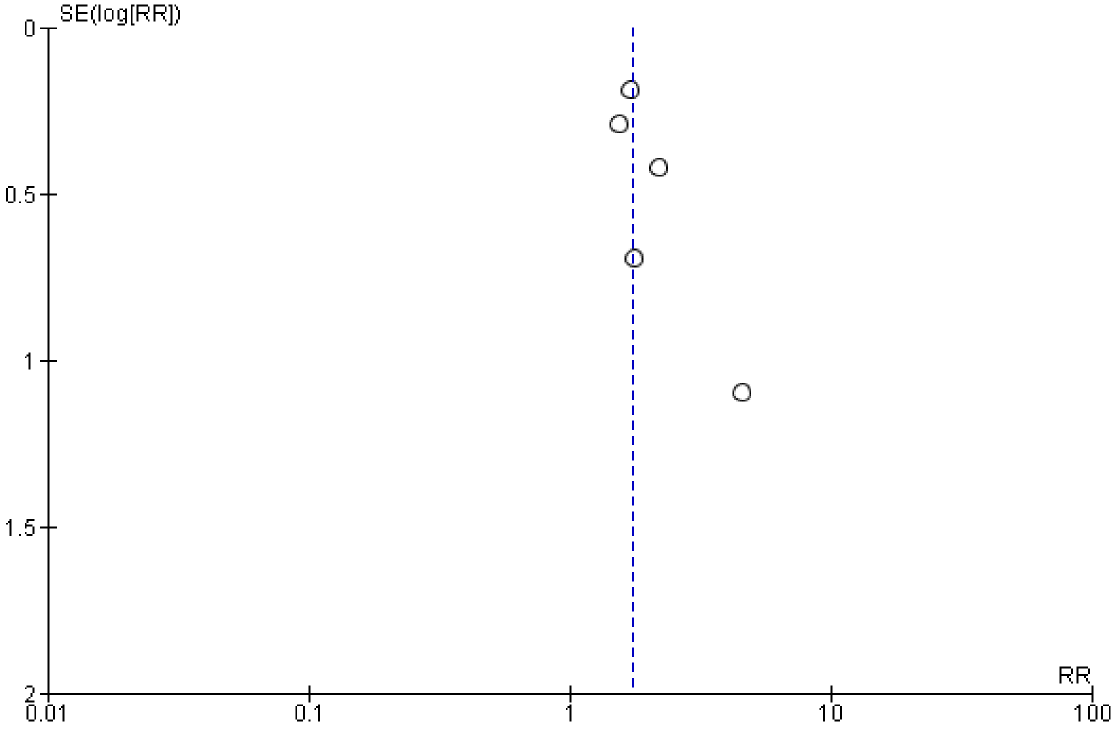

References

[1] Watanakunakorn C. and Baird I. Prognostic factors in Staphylococcus aureus endocarditis and results of therapy with a penicillin and gentamicin. Am J Med Sci 1976;1.

[2] Abrams B, Sklaver A, Hoffman T, Greenman R. Single or combination therapy of staphylococcal endocarditis in intravenous drug abusers. Ann Intern Med 1979;90:789–91. doi:10.7326/0003-4819-90-5-789.

[3] Rajashekaraiah KR, Rice T, Marsh D. Clinical significance of tolerant strains of Staphylococcus aureus in patients with endocarditis. Ann Intern Med 1980;93:796–801. doi:10.7326/0003-4819-93-6-796.

[4] Korzeniowski O, Sande MA. Combination antimicrobial therapy for Staphylococcus aureus endocarditis in patients addicted to parenteral drugs and in nonaddicts. A prospective study. Ann Intern Med 1982;97:496–503. doi:10.7326/0003-4819-97-4-496.

[5] Ribera E, Gómez-Jimenez J, Cortes E, Del Valle O, Planes A, Teresa Gonzalez-Alujas M, et al. Effectiveness of cloxacillin with and without gentamicin in short-term therapy for right-sided Staphylococcus aureus endocarditis: A randomised, controlled trial. Ann Intern Med 1996;125:969–74. doi:10.7326/0003-4819-125-12-199612150-00005.

[6] Forsblom E, Ruotsalainen E, Järvinen A. Improved outcome with early rifampicin combination treatment in methicillin-sensitive Staphylococcus aureus bacteraemia with a deep infection focus - A retrospective cohort study. PLoS One 2015. doi:10.1371/journal.pone.0122824.

[7] Thwaites GE, Scarborough M, Szubert A, Nsutebu E, Tilley R, Greig J, et al. Adjunctive rifampicin for Staphylococcus aureus bacteraemia (ARREST): a multicentre, randomised, double-blind, placebo-controlled trial. Lancet 2018;391:668–78. doi:10.1016/S0140-6736(17)32456-X.

[8] Rieg S, Ernst A, Peyerl-Hoffmann G, Joost I, Camp J, Hellmich M, et al. Combination therapy with rifampicin or fosfomycin in patients with Staphylococcus aureus bloodstream infection at high risk for complications or relapse: results of a large prospective observational cohort. J Antimicrob Chemother 2020;75:2282–90. doi:10.1093/jac/dkaa144.

[9] Ruotsalainen E, Järvinen A, Koivula I, Kauma H, Rintala E, Lumio J, et al. Levofloxacin does not decrease mortality in Staphylococcus aureus bacteraemia when added to the standard treatment: A prospective and randomised clinical trial of 381 patients. J Intern Med 2006;259:179–90. doi:10.1111/j.1365-2796.2005.01598.x.

[10] Kuehl R, Morata L, Boeing C, Subirana I, Seifert H, Rieg S, et al. Defining persistent Staphylococcus aureus bacteraemia: secondary analysis of a prospective cohort study. Lancet Infect Dis 2020;20:1409–17. doi:10.1016/S1473-3099(20)30447-3.

[11] Cheng MP, Lawandi A, Butler-Laporte G, De l'Etoile-Morel S, Paquette K, Lee TC. Adjunctive Daptomycin in the Treatment of Methicillin-Susceptible Staphylococcus aureus Bacteraemia: A Randomised Controlled Trial. Clin Infect Dis 2020. doi:10.1093/cid/ciaa1000.

[12] Grillo S, Cuervo G, Carratalà J, Grau I, Pallarès N, Tebé C, et al. Impact of beta-lactam

and daptomycin combination therapy on clinical outcomes in methicillin-susceptible *Staphylococcus aureus* bacteraemia: A propensity score-matched analysis. Clin Infect Dis 2019. doi:10.1093/cid/ciz018.

[13] Park GE, Ko JH, Cho SY, Ha YE, Lee NY, Kang CI, et al. Empirical combination of a β-lactam to vancomycin may not improve outcomes of methicillin-susceptible Staphylococcus aureus bacteraemia, compared to vancomycin monotherapy. Eur J Clin Microbiol Infect Dis 2017;36:1091–6. doi:10.1007/s10096-016-2893-4.

[14] Wan X, Wang W, Liu J, Tong T. Estimating the sample mean and standard deviation from the sample size, median, range and/or interquartile range. *BMC Med Res Methodol* 2014;14:135.
